# Case report: surgical resection of right ventricular cardiac fibroma in an adult patient

**DOI:** 10.1186/s13019-021-01514-x

**Published:** 2021-05-20

**Authors:** Hirohisa Ikegami, Anthony Lemaire, Subhashini Gowda, Billie Fyfe, Mahmoud Ali, Mark J. Russo, Leonard Y. Lee

**Affiliations:** 1 Division of Cardiothoracic Surgery, Department of Surgery, Rutgers-Robert Wood Johnson Medical School, 125 Paterson Street, New Brunswick, NJ USA; 2 New Brunswick Cardiology Group, 75 Veronica Avenue, Suite 101, Somerset, NJ USA; 3 Department of Pathology and Laboratory Medicine, Rutgers-Robert Wood Johnson Medical School, 125 Paterson Street, New Brunswick, NJ USA

**Keywords:** Cardiac tumors, Cardiac fibroma, Case report, Histology

## Abstract

**Background:**

Cardiac fibromas are rare benign cardiac neoplasms, most frequently occurring in the pediatric population; with very rare cases identified in adults. The tumors are comprised of spindled cells with myofibroblastic ultrastructural features embedded in generally collagenous and elastic stroma. The tumors are intramural in the ventricles, most commonly the left ventricle. Clinical symptoms vary by location and size of tumor and some are asymptomatic. Surgical resection is curative, but rare cases require cardiac transplantation.

**Case presentation:**

We report an asymptomatic, large, right ventricular fibroma in a 64-year-old woman. The patient underwent open incisional tumor biopsy via lower hemi-sternotomy, followed by complete tumor resection via full sternotomy a week later after confirming the tumor is benign. The tumor was resected using cardiopulmonary bypass, and the defect of right ventricular free wall was repaired using a prosthetic double-patch technique. The postoperative course was uneventful. The patient was discharged to home on day 4 post-complete tumor resection.

**Conclusion:**

This report expands the existing literature for better comprehension and detection of cardiac fibroma patients and also highlights the various imaging modalities, surgical management, and histological analysis.

## Background

Cardiac fibroma is a rare benign primary tumor with only a few hundred cases reported in the last half century [1]. The tumor is predominantly found in the pediatric population and is rare in the adult population [2, 3]. Clinical presentations vary from asymptomatic to symptomatic such as arrhythmias, congestive heart failure, or sudden death [4, 5]. The most common location of the tumor is the left ventricle (57.3%), followed by right ventricle (27.5%), and interventricular septum (17.0%) [1]. Surgical removal should be considered based on symptom, size, and location. When it is indicated, complete removal is always preferred, while, partial resection, heart transplantation, or non-surgical management is also considered [5]. The present case report describes a successful surgical resection of right ventricular cardiac fibroma in an adult patient.

## Case presentation

**Fig. 1:**
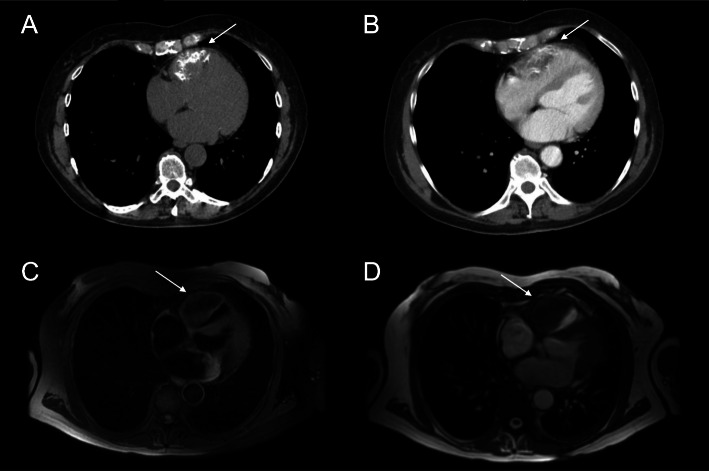
CT and MRI. **a** Non-contrast chest CT showing a partially calcified mass lesion (white arrow) associated with right ventricle. Measurement is about 4053mm. **b** Contrast chest CT showing the mass (white arrow) protruding into right ventricular cavity. **c** T1-weighted MRI image showing a soft tissue mass (white arrow) along the superior aspect of the right ventricle. **d** T2-weighted MRI image showing a low signal intensity mass (white arrow). CT: computed tomography, MRI: magnetic resonance imaging

A 64-year-old female with history of hypertension, hyperlipidemia, and arthritis was admitted to our hospital for evaluation of incidental finding of a right ventricular mass. Due to a family history of coronary artery disease and a multi-slice computed tomography (CT) coronary artery calcium score>400, she underwent left heart catheterization revealing no significant coronary artery atherosclerosis. Subsequent chest CT showed a mass lesion anterior to the right ventricle and involving the ventricular wall (Fig.1a). Cardiac magnetic resonance imaging (MRI) showed a soft tissue mass along the superior aspect of the right ventricle extending anteriorly, with a size of 4053mm (Fig. 1b). Gadolinium enhancement of the mass could not exclude malignancy.

**Fig. 2:**
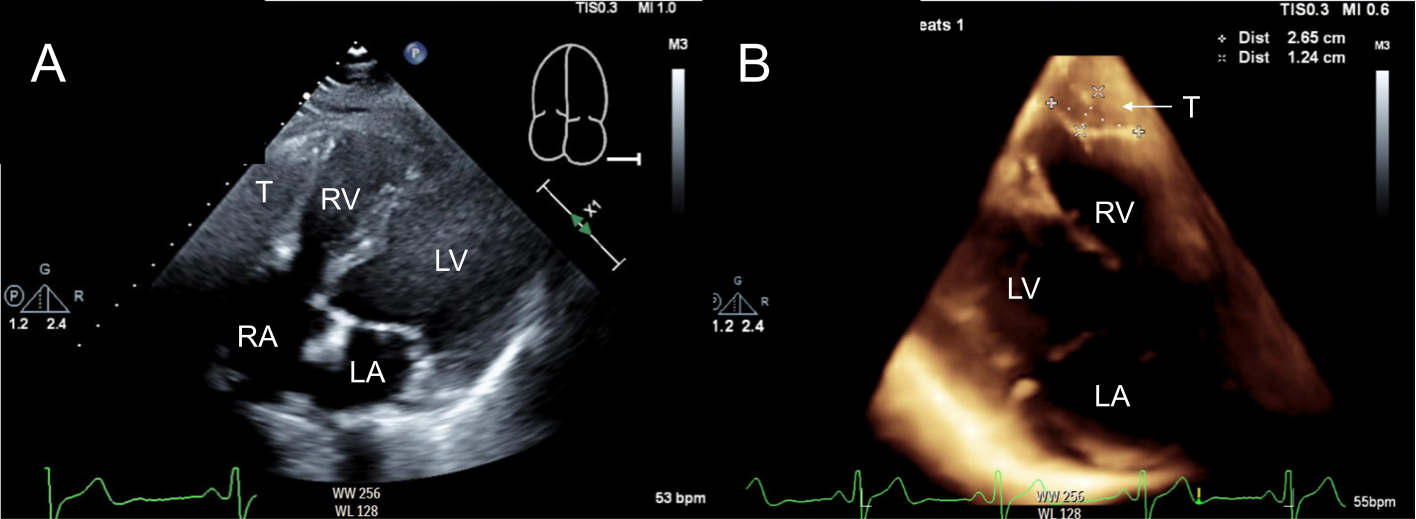
Preoperative transthoracic echocardiogram. **a** Two-dimensional four chamber view showing a non-mobile mass-like echo bright density protruding into the right ventricular free wall. The size and contiguous extension of the mass are unclear. No obvious pericardial effusion detected. **b** Three**-**dimensional view showing a well-demarcated mass in right ventricular free wall. T: Tumor, LV: Left Ventricular Cavity, RV: Right Ventricular Cavity, LA: Left Atrium, RA: Right Atrium

On admission, her physical examination was unremarkable. Vital signs were within normal limits. Routine laboratory examination was normal. A chest radiograph showed a normal heart configuration without evidence of acute cardiopulmonary pathology. Transthoracic echocardiography (TTE) demonstrated a large non-mobile echo-bright density in the right ventricular free wall (Fig.2). There was no pericardial effusion. Other cardiac structures were normal.

**Fig. 3:**
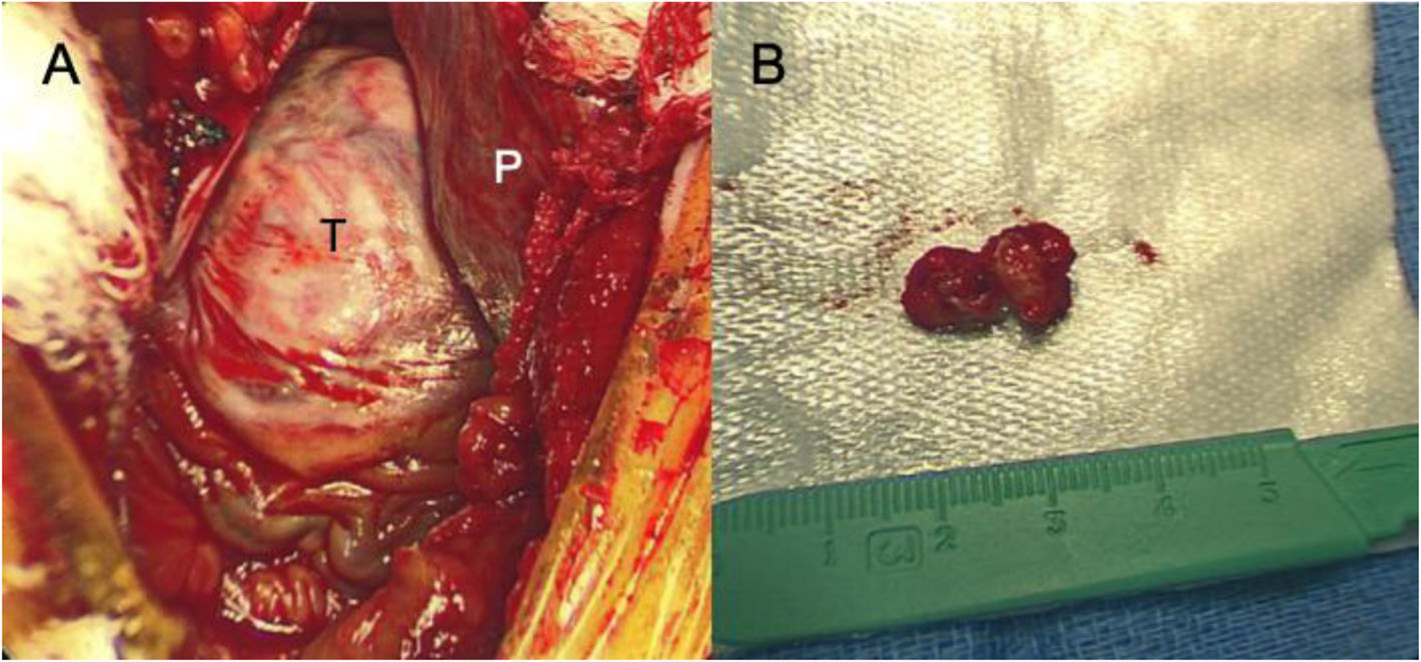
Intraoperative view at the time of incisional biopsy. **a** Post lower hemi-sternotomy view showing a surface of right ventricular free wall. Patients head is at the top. An elastic hard mass covering by white epicardium. **b** Gross appearance of biopsy specimen. T: Tumor, P: Pericardium

**Fig. 4:**
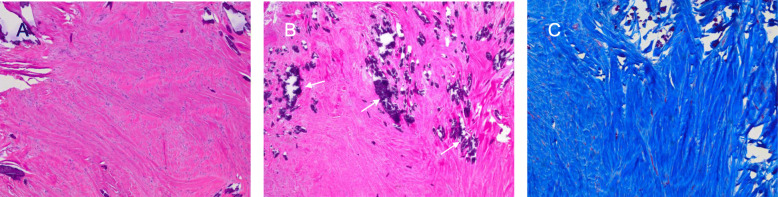
Microscopic appearance of incisional biopsy specimen. **a** The biopsy demonstrated a bland spindled cell proliferation with low overall cellularity and no mitotic activity (hematoxylin and eosin stain, 40x). **b** There was extensive calcification (white arrows) (hematoxylin and eosin stain, 100x). **c** Trichrome stain confirmed marked collagen deposition (trichrome stain, 40x)

**Fig. 5:**
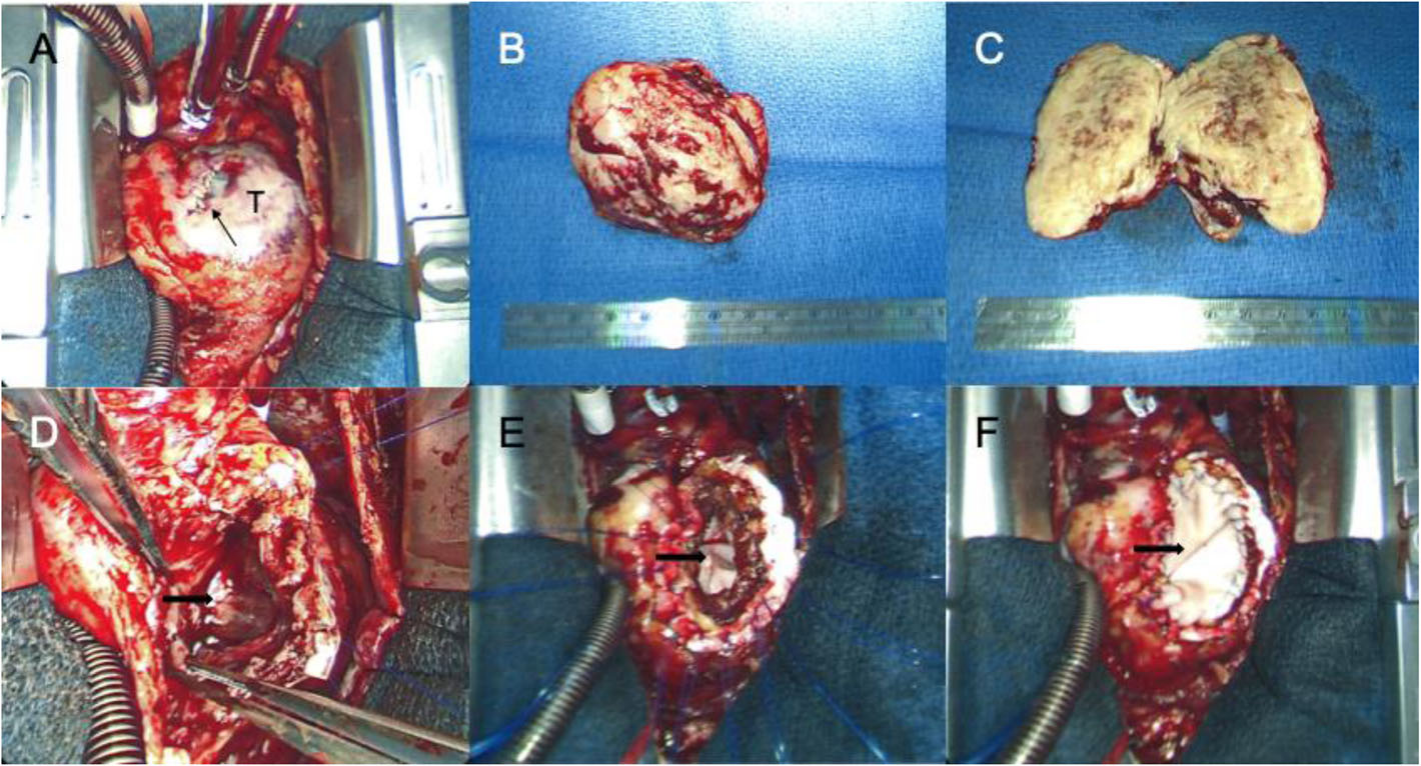
Intraoperative view at the time of complete resection. **a** Post full median sternotomy view. Patients head is at the top. Tumor covering by white epicardium. A black arrow indicates epicardia closing stitches placed at the time of incisional biopsy. **b** A resected glistening grey-red elastic hard right ventricular tumor. **c** Cut surface of excised right ventricular tumor which is gray, fibrous and trabeculated with gritty calcified specks. **d** Post tumor resection view showing a large defect in right ventricular free wall (black arrow). **e** First prosthetic patch (black arrow) sutured from right ventricular cavity side. **f** Second prosthetic patch (black arrow) placed to secure the defect closure. T: Tumor

**Fig. 6:**
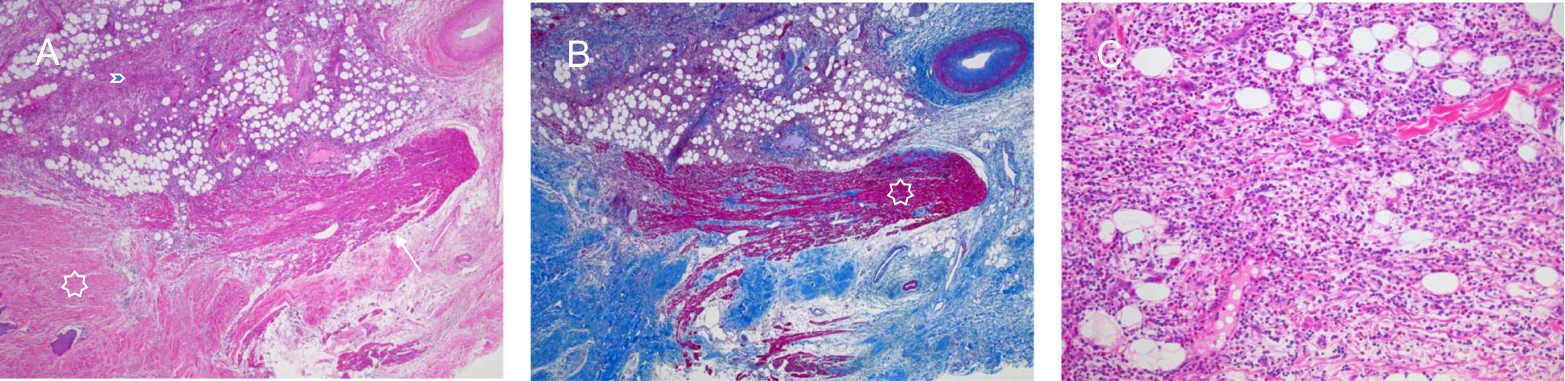
Microscopic appearance of the resected specimen. **a** The resected specimen included tumor (white star), adjacent myocardium (white arrow) and epicardium (blue arrowhead) (hematoxylin and eosin stain, 40x). **b** The tumor was not encapsulated and trapped bundles of myocytes (white star) are noted on this trichrome stain along the edge of the tumor (trichrome stain, 40x). **c** The epicardium demonstrated marked inflammation with eosinophils felt to relate to the recent surgery (hematoxylin and eosin stain, 100x)

Surgical incisional biopsy of the tumor was performed through a lower hemi-sternotomy approach (Fig.3a and b). The biopsy specimen was composed primarily of white firm tissue with a somewhat whorled appearance. Microscopically, the lesion was a spindle cell lesion with low overall cellularity. There was no noted mitotic activity and KI-67 index was zero. The nuclei of the spindled cells were bland without pleomorphism. No necrosis was noted. Marked dystrophic calcification was noted (Fig.4). The histopathological features were consistent with cardiac fibroma. She subsequently, underwent complete surgical resection of her tumor through median sternotomy under standard cardiopulmonary bypass, bicaval and aortic cannulation without aortic cross-clamping. A large glistening grey-red firm tumor (weight: 58g, dimension: 604235mm) arising from the right ventricular free wall was successfully excised with careful dissection. The outer surface was slightly lobulated. The cut surface was grayish white, fibrous and trabeculated with gritty calcified specks. Remaining extremely thin myocardial tissue of the right ventricular free wall was also excised to create a large defect (Fig.5a, b, c, and d). Tricuspid and pulmonary valves were carefully evaluated through the resultant right ventricular defect. Both valves looked intact and there was enough margin to repair the defect without compromising the valve functions. The defect was closed with double-layer patch technique by using a XenoSure biologic patch (LeMaitre Vascular, Inc., MA, USA) (Fig. 5d and f). Cardiopulmonary bypass time was 94min. The patient was easily weaned from cardiopulmonary bypass with a small amount of inotropic support. Intraoperative transesophageal echocardiogram demonstrated reasonable right ventricular function without tricuspid or pulmonary regurgitation. Microscopically, the complete resection specimen showed same characteristics as the biopsy specimen. In addition, the specimen demonstrated entrapped myocardium along the edge of the lesion and a marked epicardial inflammatory infiltrate that was rich in eosinophils suggestive of inflammatory reaction to prior biopsy, focally infiltrating the adjacent myocardium (Fig.6). Epicardial vessels in the area demonstrated mild intimal hyperplasia. The patient had an uneventful hospital course except for paroxysmal atrial fibrillation and was discharged home on postoperative day 4. Cardiac MRI on postoperative month 9 showed mild septal hypokinesis although there are limitations created by artifact from the patch and sternotomy wires. There was no enhancing mass suggesting recurrent tumor or non-enhancing thrombus in right ventricle.

## Discussion

Cardiac fibroma is a benign tumor consisting of fibroblasts in a collagenous stroma. They are generally paucicellular without mitotic activity, or necrosis and dystrophic calcification is common. These microscopic features are similar to those of fibromas arising from other parts of the body. Macroscopically, they are solitary, circumscribed, firm, gray-white, partially calcified neoplasms without a capsule. The dystrophic calcification often observed in cardiac fibroma is felt to reflect inadequate blood supply to the tumor [6].

Cardiac fibroma is mostly found in ventricles and rarely found in right atrium (5.3%) or left atrium (1.8%) [1]. They can cause intractable congestive heart failure, conduction system blockage, arrhythmia, valve dysfunction, ventricular inflow and/or outflow obstruction, coronary artery obstruction, and sudden death depending on their size and location [5]. In contrast to cardiac myxoma or fibroelastoma, cardiac fibroma scarcely causes embolic phenomenon [7]. The tumor grows in normal myocardial tissue while displacing and distorting the functioning myocardium which may result in the above various symptoms. Cardiac fibromas are occasionally hemodynamically significant by obstructing the ventricular inflow and/or outflow tracts, coronary arteries or the valves. Occasionally, the growth process involves cardiac conduction system resulting heart block or arrhythmia [4]. In our case, the tumor was found incidentally which was causing no symptom. As the tumor arose from right ventricular free wall, there was some distant from the tricuspid and pulmonary valves causing neither distortion of the subvalvular apparatus nor interference in the valvular function. Moreover, it was no relationship to major coronary circulation or conduction system. Conversely, cardiac fibroma can be a differential diagnosis for congestive heart failure, heart block, arrhythmia, valve dysfunction, and coronary obstruction.

At the present day, any one of TTE, CT, or MRI is an initial diagnostic modality depending on the initial presentation. TTE is non-invasive, fast, less-expensive diagnostic tool which does not use radiation. It can provide ventricular and valvular function in addition to tumor size, location, and surrounding structures although its sensitivity is limited by a blind spot caused by the interposition of lung, rib, and sternum. CT and cardiac MRI can provide excellent images to locate and measure the tumor with its surrounding structures. In addition, cardiac MRI shows hemodynamic and functional data. The MRI-based diagnosis demonstrates 95% accuracy to identify tumors as benign or malignant although the histological diagnosis which is considered the gold standard [8]. Coronary angiogram is usually not necessary for young patients as long as no obvious clinical sign of coronary involvement, however; it is essential to identify displaced or occluded coronary by ventricular fibromas as well as other coronary artery diseases to mitigate and stratify the risk of perioperative myocardial infarction in middle-aged and older patient population like our case. In the present study, TTE showed normal ventricular functions without valvular abnormalities. CT and cardiac MRI provided precise tumor location and size which helped in planning surgery based on their quality of studies.

Surgical intervention is recommended in symptomatic cardiac fibroma patients. We believe surgical intervention is also recommended in asymptomatic patients regardless of the size of the tumor at the time of diagnosis due to the nature of the proliferating tumor over time. Prognosis after surgical intervention is generally reported to be favorable [2, 3]. Complete surgical resection of the cardiac fibroma is the first preferable goal of surgical intervention. If the complete resection is not achievable due to an extreme risk of injury to the essential cardiac anatomy, partial resection might be a better strategy than complete resection. Cho et al. reported a case of a sufficient long-term prognosis for 14.8years after a partial resection of cardiac fibroma [5]. Others also reported favorable late results after incomplete resection of cardiac fibroma [9, 10]. Kusajima et al. reported a successful complete resection of a recurrent cardiac fibroma 21years after the initial incomplete resection of the tumor [11]. Heart transplantation remains a potential option if unrepairable cardiac damage is expected at the time of tumor resection [12]. When severe right ventricular dysfunction or major damages to cardiac structures are highly anticipated, the surgical candidacy for durable ventricular assist device or heart transplantation should be assessed preoperatively. In our case, two-stage surgery was planned because preoperative gadolinium-enhanced MRI could not rule out malignancy. Neoadjuvant chemotherapy followed by radical surgery is a safe and effective strategy in patients with primary right-side heart malignancy [13]. An intraoperative frozen section consultation would be considered to avoid two-stage surgery.

Cardiopulmonary bypass is usually necessary to perform precise tumor resection without unnecessary damage to surrounding structures, and possible intervention to cardiac valves and coronary arteries. Our case did not require aortic cross clamping and cardioplegic arrest due to the tumor location which was right ventricular free wall, while cardiac fibromas located in left side of the heart or interventricular septum require aortic cross clamping and cardioplegic arrest to obtain optimal bloodless operative field. Removal of cardiac fibroma can be performed by sharp dissection of a border between the tumor and the myocardium. This tumor does not have a capsule; however, the dissection plane is easily distinguished and a so-called surgical resection margin is unnecessary because of the nature of a benign tumor. On the other hand, other fibromas such as uterine fibromas require intraoperative determination of resection margins because they do have limited potential for malignant conversion or harbor malignancy within regions of the fibroma. Because this tumor grew in normal myocardial tissue, the tumor was not protruding into right ventricular cavity in our case. An extremely thin right ventricular myocardium was left after the tumor resection. Nevertheless, the remaining myocardium was completely resected to make a large defect for an appropriate secure reconstruction. The defect can be closed by either primary or patch repair. A primary repair is generally suitable for a small defect using Teflon felt. A patch repair is recommended for a large defect using an autologous pericardium or a prosthetic material. The benefit of the patch closure would be to prevent distortion of surrounding tissue and excessive reduction of ventricular cavity causing low cardiac output syndrome and heart failure. In our opinion, double-patch repair technique would be preferable technique for a repair of large left ventricular or interventricular septal defect to minimize the risk of bleeding or residual shunts, whereas single-patch repair technique might be sufficient for right ventricular or atrial repair since they are a low-pressure system. In our case, the defect post tumor resection was in right ventricle, however, the intraoperative decision was made to apply double-patch repair technique for secure complete defect closure (Fig. 5d, e, and f). A decision about the repair method should be made on a case by case basis. Concomitant surgery post tumor resection varies depending on the distance from the resection edge to the surrounding structures such as subvalvular apparatus and coronary artery [5].

## Conclusions

We report a surgical case of right ventricular cardiac fibroma in an adult patient. Prosthetic double-patch technique was applied to reconstruct the right ventricular defect post-tumor resection.

## Data Availability

The dataset of this care report is available from the corresponding author on reasonable request.

## Abbreviations

CT: Computed tomography
MRI: Magnetic resonance imaging
TTE: Transthoracic echocardiography

## References

[1] Torimitsu S, Nemoto T, Wakayama M, Okubo Y, Yokose T, Kitahara K, Ozawa T, Nakayama H, Shinozaki M, Sasai D, Ishiwatari T, Takuma K, Shibuya K (2012). Literature survey on epidemiology and pathology of cardiac fibroma. Eur J Med Res.

[2] Padalino MA, Vida VL, Boccuzzo G, Tonello M, Sarris GE, Berggren H, Comas JV, di Carlo D, di Donato RM, Ebels T, Hraska V, Jacobs JP, Gaynor JW, Metras D, Pretre R, Pozzi M, Rubay J, Sairanen H, Schreiber C, Maruszewski B, Basso C, Stellin G (2012). Surgery for primary cardiac tumors in children: early and late results in a multicenter European Congenital Heart Surgeons Association study. Circulation..

[3] Cho SH, Fritz T, Cronin LJ, Cohle SD (2015). Primary cardiac fibroma in an adult. Case Rep Cardiol.

[4] Chen Y, Sun J, Chen W, Peng Y, An Q (2013). Third-degree atrioventricular block in an adult with a giant cardiac fibroma. Circulation..

[5] Cho JM, Danielson GK, Puga FJ, Dearani JA, McGregor CG, Tazelaar HD (2003). Surgical resection of ventricular cardiac fibromas: early and late results. Ann Thorac Surg.

[6] Jha NK, Kiraly L, Tamas C, Talo H, Khan MD, Badaoui HE (2015). Large cardiac fibroma and teratoma in children- case reports. J Cardiothorac Surg.

[7] Elderkin RA, Radford DJ (2002). Primary cardiac tumours in a paediatric population. J Paediatr Child Health.

[8] Fussen S, De Boeck BW, Zellweger MJ, Bremerich J, Goetschalckx K, Zuber M (2011). Cardiovascular magnetic resonance imaging for diagnosis and clinical management of suspected cardiac masses and tumours. Eur Heart J.

[9] Beghetti M, Gow RM, Haney I, Mawson J, Williams WG, Freedom RM (1997). Pediatric primary benign cardiac tumors: a 15-year review. Am Heart J.

[10] Becker AE (2000). Primary heart tumors in the pediatric age group: a review of salient pathologic features relevant for clinicians. Pediatr Cardiol.

[11] Kusajima K, Hata H, Fujita T, Shimahara Y, Sato S, Ishibashi-Ueda H, Kobayashi J (2015). Successful surgical treatment for recurrent cardiac fibroma 21 years after resection. Surg Case Rep.

[12] Michler RE, Goldstein DJ (1997). Treatment of cardiac tumors by orthotopic cardiac transplantation. Semin Oncol.

[13] Abu Saleh WK, Ramlawi B, Shapira OM, Jabbari OA, Ravi V, Benjamin R (2017). Improved outcomes with the evolution of a neoadjuvant chemotherapy approach to right heart sarcoma. Ann Thorac Surg.

